# Clinical value of minimum apparent diffusion coefficient for prediction of clinically significant prostate cancer in the transition zone

**DOI:** 10.1007/s10147-023-02324-y

**Published:** 2023-03-24

**Authors:** Ashita Ono, Takeshi Hashimoto, Toshihide Shishido, Yosuke Hirasawa, Naoya Satake, Kazunori Namiki, Kazuhiro Saito, Yoshio Ohno

**Affiliations:** 1 Department of Urology, Tokyo Medical University, 6-7-1 Nishishinjuku, Shinjuku-ku, Tokyo, 1600023 Japan; 2 Department of Radiology, Tokyo Medical University, 6-7-1 Nishishinjuku, Shinjuku-ku, Tokyo, 1600023 Japan

**Keywords:** Prostate cancer, Apparent diffusion coefficient, Transition zone

## Abstract

**Background:**

This study investigated the association between apparent diffusion coefficients in Prostate Imaging Reporting and Data System 4/5 lesions and clinically significant prostate cancer in the transition zone.

**Methods:**

We included 102 patients who underwent transperineal cognitive fusion targeted biopsy for Prostate Imaging Reporting and Data System 4/5 lesions in the transition zone between 2016 and 2020. The association between apparent diffusion coefficients and prostate cancers in the transition zone was analyzed.

**Results:**

The detection rate of prostate cancer was 49% (50/102), including clinically significant prostate cancer in 37.3% (38/102) of patients. The minimum apparent diffusion coefficients in patients with clinically significant prostate cancer were 494.5 ± 133.6 µm^2^/s, which was significantly lower than 653.8 ± 172.5 µm^2^/s in patients with benign histology or clinically insignificant prostate cancer. Age, prostate volume, transition zone volume, and mean and minimum apparent diffusion coefficients were associated with clinically significant prostate cancer. Multivariate analysis demonstrated that only the minimum apparent diffusion coefficient value (odds ratio: 0.994; *p* < 0.001) was an independent predictor of clinically significant prostate cancer. When the cutoff value of the minimum apparent diffusion coefficient was less than 595 µm^2^/s, indicating the presence of prostate cancer in the transition zone, the detection rate increased to 59.2% (29/49) in this cohort.

**Conclusion:**

The minimum apparent diffusion coefficient provided additional value to indicate the presence of clinically significant prostate cancer in the transition zone. It may help consider the need for subsequent biopsies in patients with Prostate Imaging Reporting and Data System 4/5 lesions and an initial negative targeted biopsy.

## Introduction

Prostate cancer (PC) is the fourth most common cancer globally, and 1.41 million new cases were diagnosed in 2020 [1]. The National Comprehensive Cancer Network guidelines for early detection of PC recommend multiparametric magnetic resonance imaging (MRI) before biopsy [2]. Moreover, the introduction of the Prostate Imaging Reporting and Data System (PI-RADS) has improved the detection of clinically significant PC (csPC) [3]. However, the detection rate of csPC has been reported to be as low as 21.3–70.5% in PI-RADS 4 lesions and 35.6–95.0% in PI-RADS 5 lesions [4–8]. Therefore, we often encounter patients with PI-RADS 4/5 lesions and an initial negative targeted biopsy. There is a need to understand how to manage such patients. In some cases, follow-up with a repeat MRI may be a possible option, while others may need an immediate repeat biopsy.

Several reports have demonstrated that the apparent diffusion coefficient (ADC) values can be used to detect several types of cancer [8, 9]. Regarding PC, there have been reports that ADC values of cancer tissue were significantly lower than those of normal peripheral zone (PZ) tissue. Specifically, suspicious lesions in the PZ with ADC values below 750–900 µm^2^/s are more likely to represent PC [3]. However, the reference ADC value for predicting PC in the transition zone (TZ) remains unclear [10–14].

This study aims to investigate the association of ADC values in PI-RADS 4/5 lesions and csPC in the TZ of the prostate, as well as the follow-up of patients with PI-RADS 4/5 lesions in the TZ and initial negative targeted biopsy.

## Patients and methods

This retrospective study was conducted according to the ethical guidelines for clinical studies of the Ministry of Health, Labor and Welfare of Japan and approved by the Ethics Committee of Tokyo Medical University (No. T2020-0335). The need for informed consent was waived by the Ethics Committee of Tokyo Medical University.

We reviewed records of 146 patients with PI-RADS 4/5 lesions who underwent targeted transperineal prostate biopsy at Tokyo Medical University Hospital between January 2016 and July 2020. Forty-four patients with PI-RADS 4/5 lesions in only the PZ or whose medical records were insufficient for analysis were excluded from the present study. Finally, 102 patients were included in the present study.

**Fig. 1:**
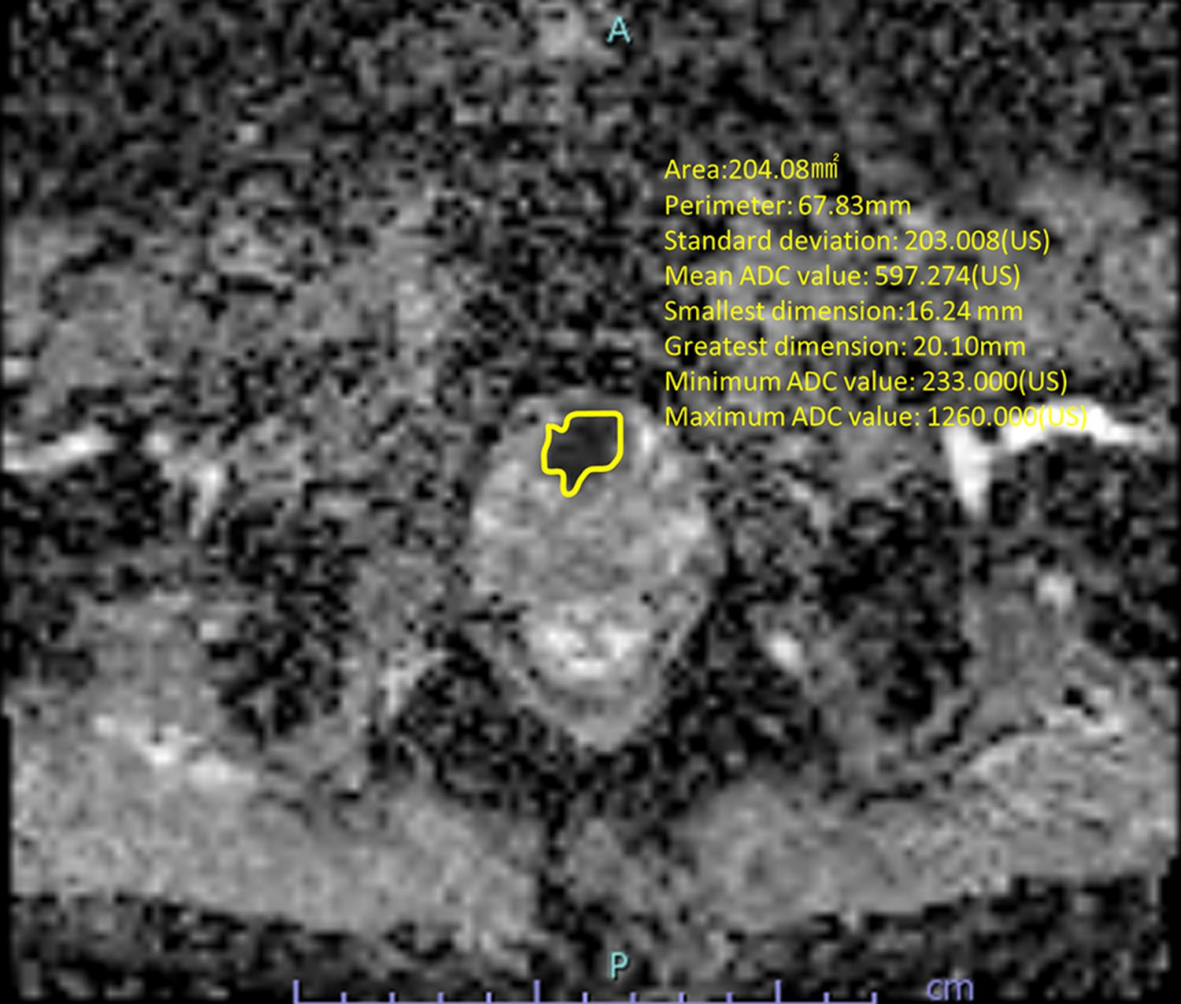
Measurement of ADC values in PI-RADS 4/5 lesion

MRI was performed using a 3.0 Tesla scanner 60-channel coil system (Skyra, Siemens, Erlangen, Germany). A bi-parametric MRI was used instead of a contrast MRI, and a unified protocol was used for all MRI examinations. Axial, coronal, and sagittal T2-weighted images were obtained, and the parameters were as follows: repetition time (TR): 3000 ms, echo time (TE): 103 ms, flip angle: 140°, slice thickness: 3 mm, resolution: 0.4 × 0.4 mm. Axial diffusion-weighted imaging was performed, and the parameters were as follows: TR: 4070 ms, TE: 76 ms, flip angle: 180°, *b*-values: 0, 800, 1500 s/mm^2^; average: 3, resolution: 0.83 × 0.83 mm. Quantitative ADC maps were created on a voxel-by-voxel basis for all b-values using the software on the scanner. The images were read by several radiologists during daily clinical practice. The PI-RADS category was assessed based on the second version of the PI-RADS [3]. The PI-RADS score obtained from the patients’ medical records was used. The ADC values for PI-RADS 4/5 lesions were calculated on an ADC map by manually tracing the target lesions (Fig. 1).

Regarding the prostate biopsy, transperineal cognitive fusion targeted biopsy using a template for brachytherapy was performed. Two to six cores were obtained by targeted biopsy for suspicious areas, and then 12-core systematic biopsy was performed.

The primary endpoint of this study was csPC in targeted biopsy specimens of PI-RADS 4/5 lesions. A Gleason score of ≥ 3 + 4 = 7 defined csPC.

### Statistical analyses

An unpaired *t* test was used to make comparisons between the two groups. Independent predictive factors for the presence of csPC were identified using the logistic regression model. From the receiver operating characteristic (ROC) curve analysis, an optimal cutoff ADC value for diagnosing csPC of the TZ of the prostate was determined using the cut point nearest to the upper left-hand corner. Based on this data-driven cutoff value, sensitivity and specificity were assessed. Statistical significance was set at *p* < 0.05. SPSS26 Statistics software (IBM, New York, NY, USA) was used for data management.

## Results

**Fig. 2:**
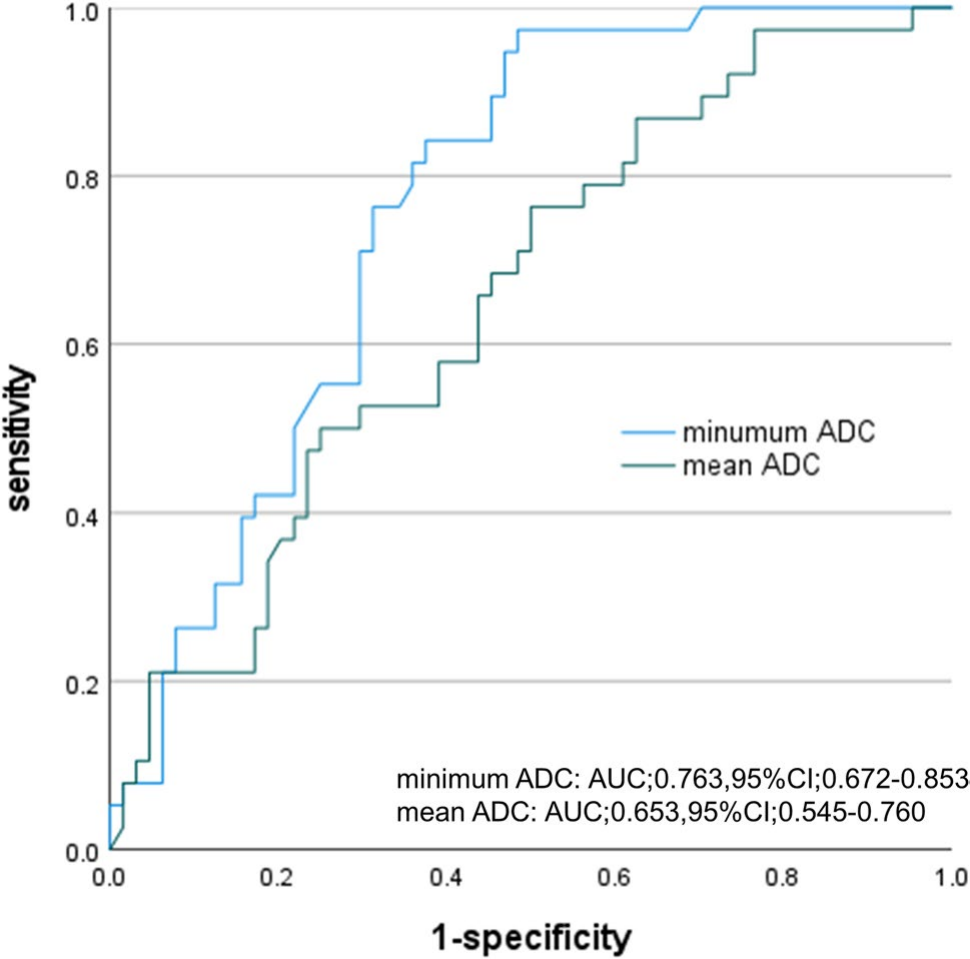
Receiver operating characteristic curve of minimum ADC values to predict clinically significant prostate cancer in the transition zone of the prostate

Patient characteristics and MRI findings are shown in Table 1. The detection rate of all PCs was 50/102 (49%), and the detection rate of csPC was 38/102 (37.3%). The presence of csPC was significantly associated with older age at biopsy, higher prostate-specific antigen (PSA) value, smaller total prostate volume, smaller transition zone volume, and longer greatest dimension of target lesion. Furthermore, the mean and minimum ADC values in patients diagnosed with csPC were 692.5 ± 150.6 µm^2^/s and 494.5 ± 133.6 µm^2^/s, respectively. These values were significantly lower than those in patients diagnosed with benign histology or clinically insignificant PC (mean: 785.9 ± 168.6 µm^2^/s, minimum: 653.8 ± 172.5 µm^2^/s). Table 2 shows the results of the univariate and multivariate analyses of the predictors for csPC. Univariate analyses demonstrated that age (*p* = 0.012), prostate volume (*p* = 0.043), transition zone volume (*p* = 0.046), greatest dimension of target lesion (*p* = 0.002), mean ADC value (*p* = 0.008), and minimum ADC value (*p* < 0.001) were significantly associated with csPC. Multivariate analysis demonstrated that transition zone volume (odds ratio [OR] 0.996; 95% confidence interval [CI] 0.939–0.994; *p* = 0.019), greatest dimension of target lesion (OR 1.267; 95% CI 1.074–1.494; *p* = 0.005), and minimum ADC value (OR 0.994; 95% CI 0.991–0.998; *p* < 0.001) were independent predictors of csPC in patients with PI-RADS 4/5 lesions. Figure 2 shows the ROC curve of the minimum ADC value for predicting csPC. The area under the curve of minimum ADC value was 0.763 (95% CI 0.672–0.853). A minimum ADC of 595 µm^2^/s was the best cutoff value for predicting the presence of csPC in PI-RADS 4/5 lesions. When the patients were divided into 2 groups by the minimum ADC of 595 µm^2^/s, 49 had a lesion with a minimum ADC < 595 µm^2^/s and 53 patients had a lesion with minimum ADC ≥ 595 µm^2^/s. The biopsy results of the two groups are shown in Table 3. The detection rate of csPC in the 49 patients with an ADC < 595 µm^2^/s was 59.2% (29/49). This rate was higher than that for all 102 patients with PI-RADS 4/5 lesions (37.5%). The discriminative powers between the presence and absence of csPC are as follow: sensitivity, 76.3%; specificity, 68.7%; positive predictive value, 59.2%; negative predictive value, 83%.

**Table 1:** Patients' characteristics and magnetic resonance imaging findings

	All cases	Benign histology and clinically insignificant cancer	Clinical significant cancer	*p* value
No. of patients	102	64	38	
Age (years old)	67.9	66.5 ± 6.9	70.4 ± 7.5	0.009
PSA (ng/ml)	11.8	10.0 ± 9.0	14.8 ± 14.6	0.04
Total prostate volume (mean, ml)	45.2	48.9 ± 22.8	38.9 ± 23.8	0.03
Transition zone volume (mean, ml)	26.8	29.8 ± 18.9	21.8 ± 18.3	0.04
PI-RADS score				
4	80 (79.4%)	54	26	0.06
5	22 (20.6%)	10	12	
ADC value (µm^2^/s)				
Mean	751.0 ± 167.6	785.9 ± 168.6	692.5 ± 150.6	0.006
Minimum	594.4 ± 176.3	653.8 ± 172.5	494.5 ± 133.6	< 0.001
Maximum	922.4 ± 225.6	922.3 ± 218.5	922.6 ± 240.1	0.99
Greatest dimension of target lesion (mean, mm)	8.0 ± 3.3	7.2 ± 2.7	9.4 ± 3.7	< 0.001
Target biopsy cores (mean ± SD)	4.3 ± 2.1	3.8 ± 1.0	5.1 ± 3.1	0.01
Results of biopsy				
No. of negative cases (%)	52 (51.0%)	52	0	
No. of positive cases (%)	50 (49%)			
GS 6	12 (11.8%)	12	0	
GS 7	29 (28.4%)	0	29	
GS 8	7 (6.9%)	0	7	
GS ≥ 9	2 (2.0%)	0	2	

*PSA* prostate-specific antigen*, PI-RADS* Prostate Imaging Reporting and Data System, *ADC* apparent diffusion coefficient, *GS* Gleason score

**Table 2:** Results of univariate and multivariate analyses

	Univariate	*p* value	Multivariate	*p* value
	Odds ratio (confidence interval)		Odds ratio (confidence interval)	
Age	1.082 (1.017–1.150)	0.012	–	–
PSA	1.041 (0.996–1.088)	0.076	–	–
Total prostate volume	0.979 (0.960–0.999)	0.043	–	–
Transition zone volume	0.974 (0.949–1.000)	0.046	0.966 (0.939–0.994)	0.019
Greatest dimension of target lesion	1.244 (1.082–1.430)	0.002	1.267 (1.074–1.494)	0.005
Mean ADC value	0.996 (0.994–0.999)	0.008	–	–
Minimum ADC value	0.994 (0.991–0.997)	< 0.001	0.994 (0.991–0.998)	< 0.001
Maximum ADC values	1.000 (0.998–1.002)	0.99	–	–

*PSA* prostate-specific antigen, *ADC* apparent diffusion coefficient

**Table 3:** Association between minimum ADC value and results of biopsy

	Clinically significant cancer	Benign histology and clinically insignificant cancer	Total
Minimum ADC			
< 595	29	20	49
≥ 595	9	44	53
Total	38	64	102

*ADC* apparent diffusion coefficient

### Follow-up using MRI in patients with PI-RADS 4/5 lesions and negative targeted biopsy

Twenty-two (42.3%) of 52 patients with negative targeted biopsy underwent follow-up MRI. Of the 22 patients, 17 (77.3%) had their lesions reclassified to PI-RADS category 3 or less. The minimum ADC value of pre-biopsy MRI of these 17 patients was 713.5 ± 169.6 µm^2^/s. Of the five patients who still showed PI-RADS 4/5 lesions on the follow-up MRI, three underwent subsequent biopsy. Two patients refused immediate biopsy and were followed up with PSA. One patient with a minimum ADC value of 219 µm^2^/s on follow-up MRI was diagnosed with csPC. The other two patients with minimum ADC values of 675 µm^2^/s and 454 µm^2^/s on follow-up MRIs showed negative subsequent biopsies.

## Discussion

The present study evaluated the association between ADC values of PI-RADS 4/5 lesions in the TZ of the prostate and detection of csPC. Although PI-RADS 4/5 lesions are thought to be suspicious or highly suspicious lesions of csPC, not all patients with PI-RADS 4/5 lesions are necessarily diagnosed with cancer through biopsy. It has been reported that the detection rate of csPC was 21.3–70.5% in PI-RADS 4 lesions and 35.6–95.0% in PI-RADS 5 lesions [4–8]. One reason for a negative biopsy in patients with PI-RADS 4/5 lesions is the false positivity of a PI-RADS assessment. Additionally, the dominant factors for PI-RADS assessment of the TZ are T2-weighted images; however, TZ cancers may be difficult to identify on T2-weighted images because the TZ is often composed of variable amounts of intermixed glandular and stromal tissues, thus demonstrating heterogeneous signal intensity [3]. Stolk et al. [15] reported that inexperienced radiologists had a significantly higher false-positive rate through PI-RADS assessment in TZ lesions than that in PZ lesions. In the present study, the percentage of downgraded PI-RADS classifications on follow-up MRI after a negative targeted biopsy was 77.3% (17/22). These patients may have had false-positive results by inter- or intra-observer variability of the radiologist and/or sub-clinical prostatitis. One possible solution to reduce the false-positive rate from the PI-RADS assessment is to refer to the ADC value. Table 4 shows recent reports on the ADC cutoff value for predicting csPC [12, 14, 16–18]. The cutoff values of the mean ADC range from 750 µm^2^/s to 830 µm^2^/s. The study by Wu et al. reported that a minimum ADC value of only 570 µm^2^/s within the tumor could differentiate between a Gleason score of 3 + 4 and 3 + 3. However, these studies evaluated the ADC cutoff values for the whole prostate without distinguishing the zonal location of the prostate. It is important to make a distinction as different optimal ADC cutoff values may exist between the PZ and TZ. This could be due to the ADC values of benign and malignant lesions in the TZ having been reported to be lower than those in the PZ [18–20]. To date, no study has evaluated the association between ADC values and the presence of csPC in the TZ. The present study is the first to demonstrate that the minimum ADC value in PI-RADS 4/5 lesions is an independent predictor for csPC in the TZ and to specify that a minimum ADC of 595 µm^2^/s is the best cutoff value for predicting the presence of csPC in PI-RADS 4/5 lesions. When the indication for targeted biopsy was set for lesions with a minimum ADC < 595 µm^2^/s, only 49 patients underwent biopsy. The detection rate of csPC was 29/49 (59.2%), as shown in Table 3. This rate is higher than that for all 102 patients with PI-RADS 4/5 lesions (37.5%). On the other hand, 53 patients with a minimum ADC ≥ 595 µm^2^/s did not undergo biopsy. Therefore, 44 (68.7%) of 53 patients can avoid unnecessary biopsy. Instead, csPC was missed without biopsy in 9 (23.7%) of 53 patients. We hypothesize that the use of the minimum ADC value improves the detection rate of csPC and decreases the number of unnecessary biopsies. Therefore, the minimum ADC should be added as a criteron to the PI-RADS system. However, several problems still need to be addressed, such as the different field strengths of MRIs or the differences caused by the use of MRI machines from different vendors. Thus, our results should be validated further, taking into consideration these issues, in a larger population group.

**Table 4:** Studies on apparent diffusion coefficient cutoff vaue on predict clinically significant cancer

Year	Author	No. of patients	Mean apparent diffusion coefficient values (µm^2^/s)								Cutoff value (µm^2^/s)	No. of References
			Normal tissue			Cancer						
			Whole	PZ	TZ	Whole	PZ	TZ	Non-csPC	csPC	Value for csPC (AUC)	
2016	Kim TH	125	–	–	–	NA	–	–	924	724	830 (0.88)	[16]
2017	Pepe P	44	–	–	–	639	–	–	GS 3 + 3: 750	GS 3 + 4: 635 GS 4 + 3: 489 GS 4 + 4: 485 GS 4 + 5: 498	747 (0.800)	[14]
2020	Moraes MO	91	–	–	–	–	–	–	880	750	750 (0.810)	[12]
2020	Meyer HJ	1633							1100	860	750	[17]
2017	Wu X	30	1400	1440	1330	790	820	740	830	770	–	[18]
			min 1180	min 1210	min 1120	min 570	min 590	min 530	640	540	min 570 (0.765)	

*PZ* peripheral zone, *TZ* transition zone, *csPC* clinically significant prostate cancer, *AUC* area under the curve, *min* minimum

Although many studies have evaluated the association between ADC values and detection of prostate cancer, most have focused on the mean ADC value in suspicious lesions. Moreover, studies on liver and breast tumors reported that mean and minimum ADC values were valuable for differentiating between malignant and benign lesions and that the minimum ADC value was more sensitive and specific than the mean ADC value [21, 22]. These studies could confirm the validity of our results. In addition, the ADC values were calculated on an ADC map by manually tracing the target lesions in the present study. We think that minimum ADC values are easier to use in daily clinical practice because minimum ADC values, which are the lowest ADC values within target lesions, may be less affected by manual tracing of target lesions compared to mean ADC values.

Regarding limitations, the possibility of sampling errors in targeted biopsies must be considered. The cancer detection rate by transperineal cognitive fusion targeted biopsy for lesions classified as PI-RADS 3 or greater has been reported to be 66.3–75% [23–25]. Our cancer detection rate of 49% for PI-RADS category 4/5 lesions seems to be relatively low, although there might be a subset of patients with false-positive results in the PI-RADS assessment in the present study. Additionally, while it has been reported that the cancer detection rate in TZ lesions was lower than that in PZ lesions [6, 7], the relatively low cancer detection rate in the present study might be caused by a sampling error in targeted biopsy.

To date, there have been three studies on follow-up in patients with PI-RADS 4/5 lesions whose biopsies showed benign histology. First, Hauth et al. [26] followed 26 patients with PI-RADS 4 lesions and a negative initial core biopsy. The minimum ADC values at baseline and follow-up MRI in patients with PI-RADS 4 lesions were 539 ± 180.3 µm^2^/s and 523 ± 254.8 µm^2^/s, respectively. Upon follow-up MRI, 2 lesions were downgraded to PI-RADS 3, 8 lesions remained PI-RADS 4, and 16 lesions progressed to PI-RADS 5 classification. The rate of malignancy in the second core biopsy of PI-RADS 4 lesions was 75% (18/24). An instant repeat biopsy for PI-RADS 4 lesions with an initial negative biopsy result was recommended. Second, Ullrich et al. [27] analyzed 193 patients with PI-RADS 4 lesions. In their study, the detection rate of all PCs was 119/193 (62%), and the detection rate of csPC was 92/193 (48%). However, PI-RADS 4 lesions in the TZ with overlaying signs of stromal hyperplasia showed PC in only 11% of patients (4% csPC). Therefore, they recommended repeat targeted biopsy for PI-RADS 4 lesions in the PZ and follow-up using MRI for PI-RADS 4 lesions in the TZ with overlaying signs of stromal hyperplasia. Third, Meng et al. [28] analyzed 88 patients with PI-RADS 4/5 lesions and nonmalignant pathological findings on initially targeted prostate biopsy. Of the 45 patients who underwent follow-up MRI, 73% (33/45) were downgraded to PI-RADS 3 or lower, and 27% (12/45) had persistent PI-RADS 4/5. On repeat MRI-targeted biopsy, cancer was found in 62.5% of men with PI-RADS 4/5. Therefore, they recommended prompt repeat biopsy in patients who showed PI-RADS 4/5 lesions on follow-up MRI.

In the present study, we demonstrated that a minimum ADC of 595 µm^2^/s was the best cutoff value for predicting the presence of csPC in PI-RADS 4/5 lesions in the TZ. Therefore, we propose the following follow-up protocol: When the minimum ADC value on follow-up MRI in PI-RADS 4/5 lesions in the TZ is 595 µm^2^/s or less, prompt repeat biopsy is recommended. Conversely, when the minimum ADC value is more than 595 µm^2^/s, it might be possible to substitute further follow-up using MRI for prompt repeat biopsy.

While the present study provided novel information about PI-RADS assessment in patients with PC in the TZ of the prostate, it has several limitations. First, this study was retrospectively conducted at a single institution; thus, the number of cases was relatively small. Second, there was no uniform follow-up protocol after a negative biopsy. Third, the possibility of false positivity in the PI-RADS assessment and sampling error in targeted biopsy cannot be ruled out. Because the present study analyzed data obtained during daily clinical practice, several radiologists were involved in PI-RADS assessment and multiple urologists performed cognitive fusion targeted biopsy. These conditions could be associated with false-positive results in PI-RADS assessments and sampling errors in biopsies. Fourth, PI-RADS 4 lesions in TZ consist of two different entities. Those that were scored to be PI-RADS 4 based on T2WI alone, and those that showed PI-RADS 3 feature by T2-weighted images and then upgraded to PI-RADS 4 based on diffusion-weighted image findings. These two entities were not differentiated separately in this study. This may cause bias in analyzing the association between ADC value and the presence of csPC in PI-RADS 4 lesions. Despite these limitations, we believe that the findings of the present study will contribute to more appropriate follow-up in patients with PI-RADS 4/5 lesions and initial negative targeted biopsy. Future prospective studies or analyses using whole prostatectomy specimens are warranted to validate the optimal cutoff value of the minimum ADC value of PI-RADS 4/5 lesions.

## Data Availability

The datasets used and analyzed during the current study are available from the corresponding author on reasonable request.

## Declarations

### Informed concent

The need for informed consent was waived by the Ethics Committee of Tokyo Medical University.
